# Hemophagocytic lymphohistiocytosis during pregnancy: a review of the literature in epidemiology, pathogenesis, diagnosis and treatment

**DOI:** 10.1186/s13023-021-01790-9

**Published:** 2021-06-21

**Authors:** Lidong Liu, Yutong Cui, Qiongjie Zhou, Huanqiang Zhao, Xiaotian Li

**Affiliations:** 1 Obstetrics and Gynecology Hospital of Fudan University, Fangxie Road 419, Huangpu District, Shanghai, 200011 China; 2 The Institute of Biomedical Science, Fudan University, Shanghai, China; 3 Shanghai Key Laboratory of Female Reproductive Endocrine-Related Diseases, Shanghai, China

**Keywords:** Diagnosis, Hemophagocytic lymphohistiocytosis, Infection, Pregnancy, Treatment

## Abstract

Hemophagocytic lymphohistiocytosis during pregnancy is rare; it is often misdiagnosed, resulting in a high maternal and foetal mortality rate. Herein, based on limited case reports including antepartum and postpartum cases, we reviewed the current studies of pregnancy-related hemophagocytic lymphohistiocytosis, and compared the epidemiology, aetiology, diagnosis and treatment of pregnancy-related hemophagocytic lymphohistiocytosis with non-pregnancy, enriching the understanding of hemophagocytic lymphohistiocytosis and its treatment in obstetrics.

**Supplementary Information:**

The online version contains supplementary material available at 10.1186/s13023-021-01790-9.

## Introduction

Hemophagocytic lymphohistiocytosis (HLH) during pregnancy is rare and it is often misdiagnosed, resulting in a high mortality rate. HLH is a complex disease with rapid onset, whose severe condition, diagnosis, and treatment are characterized by tissue cell proliferation, hyperinflammation, bone marrow hemophagocytic activity, and large amounts of inflammatory cytokines released by lymphocytes [1–5]. These characteristics are partly similar to those of pregnancy and related diseases such as preeclampsia. HLH is considered grossly underestimated and has attracted increasing attention because of its non-specific clinical manifestations, which are difficult to diagnose and life-threatening to the foetus [6]. There are many problems with the interaction between HLH and pregnancy, and its course during pregnancy, as well as its diagnostic characteristics and treatment need to be clarified.

Due to its low incidence and lack of clinical trials, most HLH cases have been reported in isolation. Only a few researchers have reported some causes of HLH during pregnancy and the effectiveness of treatment with steroids alone or with etoposide/cyclosporin A [7, 8]. However, there is no consensus on the diagnosis and treatment of HLH in pregnant women.

**Fig. 1:**
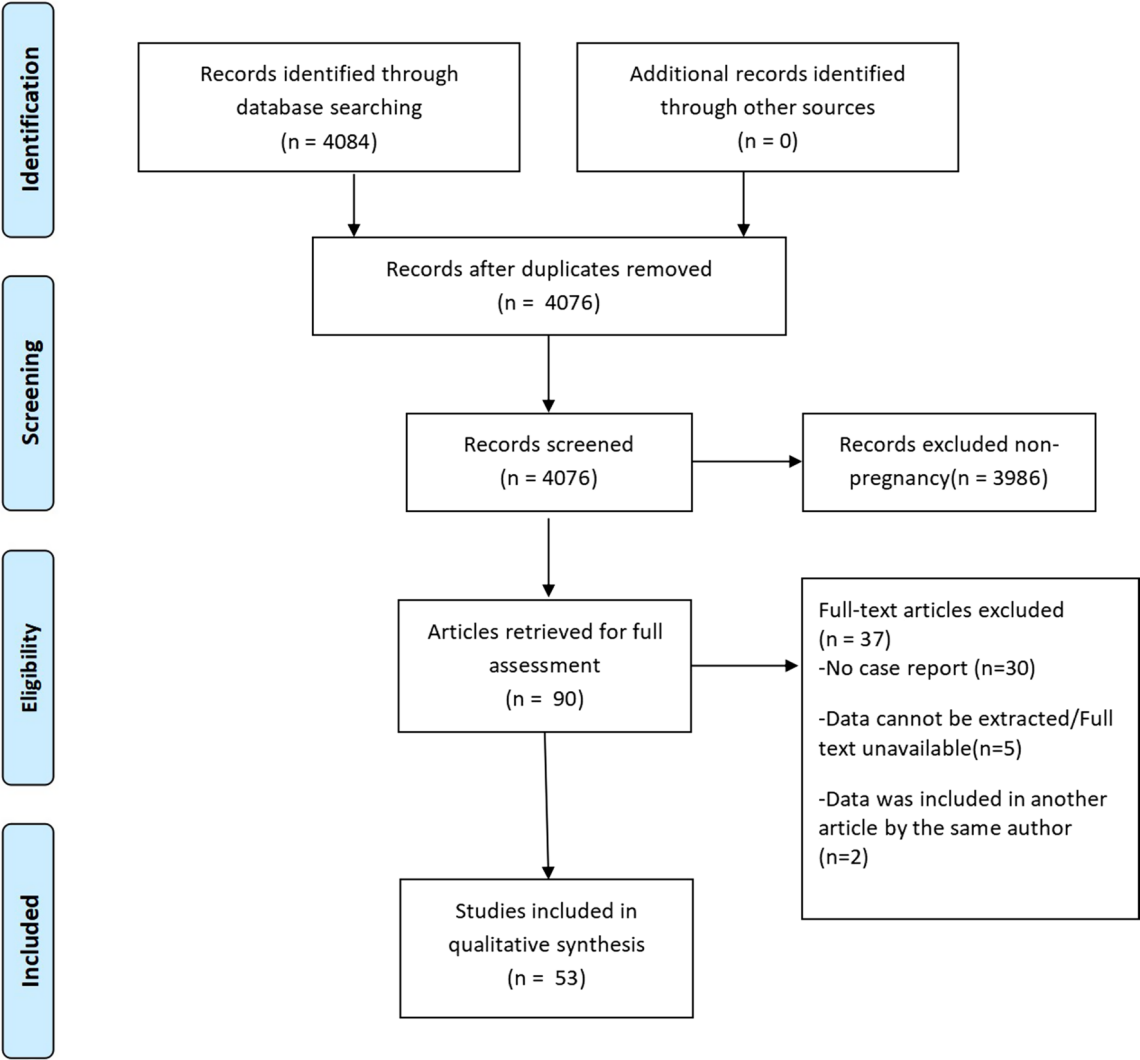
Search strategy to identify publications in this systematic review

Therefore, we conducted this retrospective review to clarify the characteristics of HLH during pregnancy in order to propose diagnostic and management principles of HLH during pregnancy. We searched “hemophagocytic lymphohistiocytosis” OR “pregnancy” as keywords and found 4084 eligible articles from the PubMed, CNKI, EMBASE, the Cochrane library databases, and 53 studies (1.3%) that were published from 1958 to 2020. Most of the data obtained were published in English and Chinese, and the full text of the reports were screened for inclusion/exclusion into the study. Literature whose data were duplicated, non-pregnancy, could not be extracted, or were not available in their entirety was excluded. After excluding interference and screening, we enrolled 81 patients (Fig. 1).

## Main text

### Pathogenesis

The pathophysiology of pregnancy-related HLH remains unclear. It may be that obstetricians do not know enough about HLH during pregnancy, and the aetiology examination of HLH patients during pregnancy is not sufficiently standardized. Among the 81 cases examined, 51 demonstrated clear etiology. Similar to non-pregnancy, infection (33/81) remained the primary factor in HLH during pregnancy, accounting for 41% of all pathogenic factors; other causes included malignancy (3/81) and genetic factors (1/81).

**Fig. 2:**
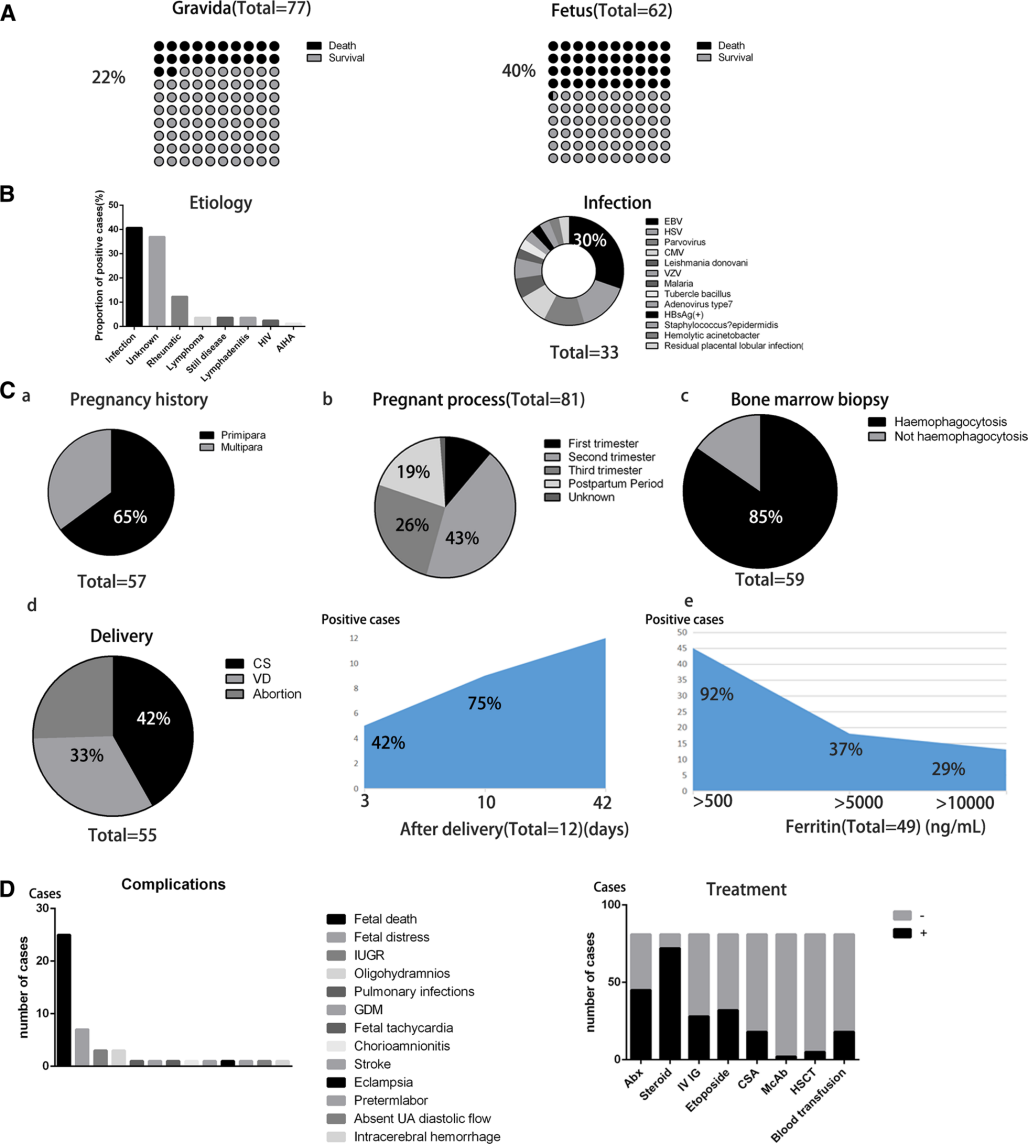
Pathogenesis, diagnosis and treatment of HLH during pregnancy. **a** In the case of HLH during pregnancy, maternal death and foetal death rate is shown by 10 × 10 dot plot. **b** (left) Proportion of different causes of HLH during pregnancy. (right)The proportion of specific pathogens in the factor of infection. **c**. **a** Comparison of HLH in the history of pregnancy between primiparas and pluripara. **b** The proportion of HLH during pregnancy in different trimesters. **c** Proportion of hemophagocytosis in 59 patients who underwent bone marrow biopsy. **d** (Left) A variety of ways of delivery. (Right) The proportion of HLH occurring on different days after delivery. **e** Number of cases of ferritin in different grades and the corresponding proportions. **d** (left) Types of complications. (right) Differences in the use of medication in HLH during pregnancy

Infection is a major factor in pregnancy-related HLH, similar to non-pregnancy-related HLH. The Epstein–Barr virus (EBV) is the major pathogen in non-pregnancy HLH. Similarly, the EBV is the primary pathogen in pregnancy-related HLH, in pregnancy-related HLH, accounting for 30% of all infection factors. Among the 81 patients, 46 had an EBV test, and 10 were positive. Therefore, for maternal HLH, EBV should be promptly identified and treated. Although the exact mechanism whereby EBV leads to HLH is unknown, it is considered that EBV disrupts the normal function of CD8+ T cells, leading to specific cytotoxic pathways in HLH during pregnancy [9]. Other infection-associated factors, including herpes simplex virus (HSV), parvovirus, cytomegalovirus, leishmania donovani, varicella zoster virus, malaria, tubercle bacillus, adenovirus type7, hepatitis B surface antigen (+), *Staphylococcus* epidermidis, Acinetobacter haemolyticus, and residual placental lobular infection, are shown in Fig. 2b.

Malignancy in pregnant women is very rare because they are usually young. However, malignancy was evident in 3.7% of the patients with HLH, suggesting that it may be the first symptom of hematologic tumours in pregnancy. The incidence rate of lymphoma and acute leukemic disease, the most common blood tumour diseases during pregnancy, are 1:6000 and 1:75,000, respectively [10], whereas transplacental transmission of maternal malignant cells is reported to be uncommon, possibly due to the placenta’s protective effect [11]. Seven cases of maternal malignant lymphoma involving the placenta were reported [12–18]. Therefore, once a mother is diagnosed with HLH, the possibility of lymphoma and other tumours must be ruled out because the prognosis of malignant tumours is poor, and early treatment is required.

Although genetic factors in pregnancy-related HLH accounted for only a small proportion of cases, they are an important cause of HLH in non-pregnancy. However, it is difficult to draw a conclusion because only one case of genetic pregnancy-related HLH was reported. Even though genetic screening and diagnosis are still very important, especially for recurrent HLH, some hospitals in developing countries cannot perform these inspections. In this study, we found that pregnant women usually do not undergo genetic testing. Only four patients underwent genetic testing, of which only one was found to be positive: a 40-year-old woman who had a novel exon 19, c.1607G>T (p.Arg536Leu) heterozygous mutation of the *UNC13D* gene, triggered during each first trimester of her first and second pregnancies [19]. Although the genetic factor for HLH during pregnancy has not received much attention, genetic examinations are recommended as soon as conditions permit.

Finally, there is a clear interaction between pregnancy and HLH. The incidence of HLH varies in terms of gestational age, number of births, and the severity of the disease. In addition, HLH can appear in any stage of pregnancy, including the antepartum (80%) and postpartum (20%) stages. In the antepartum stage, HLH mainly occurs in the second (43% in all) and third (26% in all) trimesters of pregnancy. Whether it is associated with changes in immune function during pregnancy remains unclear. In the postpartum stage, nearly half of the cases occurred within three days, and three-quarters occurred within ten days after delivery, which may be related to fluctuations in the maternal physiological state after delivery and puerperal infection (Fig. 2c). Furthermore, the incidence rate in primipara (37/81) mothers was slightly higher than that in multipara (20/81) mothers. The probable cause is maternal and foetal immunity. Contrary to non-pregnant cases, it is worth noting that 37% of all pathogenic factors were unexplained in cases of HLH during pregnancy. Pregnant women with HLH of unknown cause are difficult to treat effectively, and, more seriously, they account for a large proportion of cases. Moreover, the pregnancy itself may be a contributor, as evidenced cases that were completely relieved after pregnancy [20].

The placenta produces most cytokines; further, pregnancy itself is a systemic inflammatory response, and preeclampsia is considered a systemic inflammatory disease. Inflammatory responses and cytokine storms may induce or exacerbate HLH. Furthermore, HLH symptoms might be related to elevated β-hCG [19]. Teng et al. [21] hypothesized that the mechanism of HLH may be similar to that of preeclampsia, and that placental transport and its cytokine storm are key factors in the development of HLH during pregnancy. Additionally, similar to pregnancy-related HLH, the immature placenta releases syncytiotrophoblast components, foetal derived soluble RNA and DNA, and cytotrophoblast cells, which enter the maternal circulation, causing various immune disorders and systemic inflammatory responses [22, 23]. Therefore, it is reasonable to believe that pregnancy itself may be a factor for pregnancy-related HLH. However, there is no clinical basis and further evidence should be collected in the future.

On the other hand, HLH could have an impact on pregnancy. HLH increases the likelihood of complications during pregnancy, such as preeclampsia, postpartum haemorrhage and adverse perinatal outcomes. The possible mechanisms are as follows: (1) the influence of HLH; (2) the effect of HLH inducement on pregnancy, such as infection and rheumatic diseases; and (3) complications of HLH, such as shock and disseminated intravascular coagulation (DIC). Nevertheless because of the low incidence and insufficient epidemiological data, more insight into pregnancy-related HLH is needed.

### Diagnosis

This study comprised 53 studies and 81 pregnancy patients with a median age of 29 years (range 20–44 years). Due to the non-specific clinical features of HLH, prompt diagnosis is often challenging. Early diagnosis of HLH during pregnancy and the timely initiation of treatment is necessary to ensure maternal and foetal safety; delayed treatment may lead to missed opportunities for optimal treatment and irreversible multi-organ failure. However, due to the low incidence of pregnancy-related HLH, the diagnosis of HLH during pregnancy is still based on the HLH 2004 standard. Based on a previous analysis, a clinical diagnosis should be made, taking into consideration the ten major initial symptoms during pregnancy, which include fever, splenomegaly, hepatomegaly, jaundice, body aches, upper respiratory symptoms, fatigue, lymphadenopathy, pruritic rash, and vomiting. Serum ferritin testing is necessary, while cytokine tests can be performed conditionally. Hemophagocytic syndrome was found by bone marrow tests, and highly suspected patients need to be double-checked. In addition, a diagnostic score method has also been reported. Fardet et al. [24] verified a diagnostic score for secondary HLH. Its system includes the aetiology, organ, hyperferritinaemia, hypertriglyceridaemia, and hemophagocytosis. The web-based Delphi study also validated related diagnostic variables [25]. In this study, we found that the first symptoms of pregnancy-related HLH were non-specific and included persistent or intermittent fever (69%), which lacked specificity, followed by splenomegaly (42%), jaundice (20%), body aches (17%), upper respiratory symptoms (16%), fatigue (16%), lymphadenopathy (14%), pruritic rash (11%), and vomiting/nausea (11%). Furthermore, pregnant women usually presented to the hospital with normal blood pressure and heart rate, which worsened as the disease progressed.

Among the 81 subjects, 59 underwent biopsies (54 showed signs of hemophagocytosis and 5 were negative), and the remaining 22 were unreported. Multiple biopsy sites were reported, including the bone marrow (50 positive cases in 59 biopsy cases), spleen (2 positive cases), jejunum (1 positive case), and liver (2 positive cases). Hemophagocytosis was found in 13 patients after the second bone marrow biopsy. This lack of sensitivity indicates that hemophagocytosis does not necessarily occur in the bone marrow; rather, it may appear in any tissue other than bone marrow and does not require a positive bone marrow biopsy for diagnosis. It is important to note that a positive bone marrow biopsy may not be detected early in the course of the disease; thus, a second bone marrow biopsy could be considered.

The positive rates of hypertriglyceridemia and hypofibrinogenemia were 49% and 19%, respectively, which is half of the rate seen in non-pregnancy patients with HLH. People with ferritin levels above 500 ng/mL, 5000 ng/mL, and 10,000 ng/mL accounted for 92%, 37%, and 27%, respectively, similar to those found in non-pregnancy, as shown in Fig. 2c.

In terms of severe clinical manifestations, inadequate attention was paid to the central nervous system symptoms during pregnancy. There were eight cases of DIC and four women who demonstrated central nervous system symptoms, among whom only one underwent brain magnetic resonance imaging.

The maternal and foetal mortality rates were as high as 22% (17/77) and 40% (25/62) in the patients with available data, respectively, as shown in Fig. 2a. Foetal death accounted for the largest percentage of obstetric complications, as shown in Fig. 2d. Other complications included foetal distress, intrauterine growth retardation, oligohydramnios, pulmonary infections, gestational diabetes mellitus, foetal tachycardia, stroke, eclampsia, preterm labour, absent umbilical artery diastolic flow, and intracerebral haemorrhage.

### Differential diagnosis

The diagnosis and management of HLH during pregnancy is complex. Due to the different biological factors and therapeutic methods that overlap between the haemolysis, elevated liver enzymes, low platelet count (HELLP) syndrome; thrombotic thrombocytopenic purpura (TTP); hemolytic uremic syndrome (HUS); and HLH, it is necessary to make an accurate diagnosis [26]. Pregnancy-related HLH has clinical manifestations similar to the HELLP syndrome, such as haemolytic anaemia, elevated liver enzymes, and thrombocytopenia [27]. However, most cases of HELLP do not present with fever and hemophagocytosis changes, and its symptoms subside within a few days after delivery, whereas HLH progresses gradually [28]. Furthermore, preeclampsia is not an indicator of differentiation. Although HELLP is a complication of preeclampsia, some HELLP patients show atypical preeclampsia manifestations. Besides, HLH pregnant women can also develop preeclampsia as reported by Yamanaka et al. [29]. Thrombotic microangiopathy diseases, such as TTP, HUS, and HLH are highly correlated in clinical and laboratory tests, making them difficult to distinguish, as all patients displayed indicators, such as thrombocytopenia and anaemia. HUS is usually confined to the postpartum period, with initial signs and symptoms of renal failure. TTP usually presents with neurological impairments, such as visual impairment, epilepsy, and aphasia [30–32]. However, they demonstrated no hemophagocytosis or serum ferritin changes. Among TTP, HUS and HLH, pregnancy-related HLH has the higher mortality rate and is likely to worsen after delivery.

### Treatment

Delaying treatment of HLH during pregnancy while blood and imaging results are pending can be harmful and lead to irreversible multi-organ failure. Therefore, clinicians should initiate treatment in patients suspected of hemophagocytosis and with unexplained cytopenia and fever. Current treatment options for HLH during pregnancy include general treatment, obstetric treatment, monoclonal antibody, and hematopoietic stem cell transplantation.

#### General treatment

The severity of these patients’ inflammatory storms requires immediate treatment of the inflammation. Treating the underlying cause, without attending to the inflammation, may result in missed treatment opportunities and disease progression. Anti-inflammatory drugs include corticosteroids and chemotherapeutic drugs, such as etoposide and cyclosporin A.

The most common and relatively safest treatment during pregnancy is high doses of corticosteroids, classified as Class C by the Food and Drug Administration (FDA), which are inactivated in the placenta and associated with a relatively low risk of birth defects. We found that 89% of the patients in our study were treated with corticosteroids, usually as an initial treatment for their anti-inflammatory effects. Multiple studies [8, 33, 34] have reported cases in which HLH during pregnancy was successfully treated with high-dose corticosteroids alone, suggesting that the benefits of these drugs far outweigh the risks. Since high-dose corticosteroids have been used safely in pregnancy, corticosteroids were the only treatment used in 47% of them, excluding the use of antibiotics. Therefore, corticosteroids are the first choice to control life-threatening hyperinflammation.

Etoposide is widely applied in non-pregnant HLH patients because it is the preferred chemotherapy in the treatment regimens of HLH-1994 and HLH-2004 [2, 3, 35]. However, due to the teratogenic nature of etoposide, there are concerns regarding its use in pregnant women [36]. Limitations of etoposide, classified as Class D by the FDA, in pregnancy-related HLH may be related to foetal toxicity and strong bone marrow suppression. Stefansdottir et al. [37] showed potential adverse effects on mouse foetal ovarian development. However, studies by several scholars [7, 38, 39] have shown that the use of etoposide would be of more benefit to the patient than harm to the foetus. Song et al. [40] suggested that achieving a balance between effective treatment and foetal safety was critical. Etoposide should be used actively but at appropriate doses and low toxicity. Women who received etoposide for HLH during pregnancy had a low recurrence rate and good long-term prognosis. In our study, etoposide was used in 32 of the 81 patients, among whom 23 women were in remission after treatment, as shown in Fig. 2d. Therefore, we recommend etoposide for severe, or steroid-ineffective cases of pregnancy-related HLH.

Cyclosporin A is also an important therapeutic agent in HLH-2004 and is classified as class C by the FDA, suggesting that it is safe for the foetus. Yamaguchi et al. [41] reported that cyclosporin A was a safe and available strategy for corticosteroid-resistant women during pregnancy. Cyclosporin A had a significant effect on the foetus without intrauterine distress or growth restriction. Intravenous injection of cyclosporin A can improve clinical outcomes [42]. In our study, cyclosporin A was used in 18 of the 81 patients, among whom 13 women were in remission after treatment, as shown in Fig. 2d. Therefore, cyclosporin A is a good choice for the treatment of HLH during pregnancy.

As pregnancy-related HLH disease has a variety of aetiologies, removing the triggers is as important as controlling the inflammatory storms. Therefore, aetiological therapies are highly effective after the control of HLH acute inflammation, including R-CHOP for B-cell lymphoma [43], acyclovir for HSV infection [44], and HAART for human immunodeficiency virus infection [45]. Song et al. [40] reported that two patients who were screened for aetiological cause showed long-term survival after treatment, emphasizing the importance of determining the underlying factors for treatment.

#### Obstetric treatment

Obstetric management is imperative to ensure the safety of HLH mothers and foetuses. Close monitoring of foetuses and pregnant women, including prenatal screening (such as foetal ultrasound and electronic foetal monitoring), preparing blood products before delivery, terminating pregnancy, preparing to rescue pregnant women and newborn babies after delivery, and postpartum follow-up are vital components of obstetric management. While the termination of pregnancy is an important mean of obstetric treatment, HLH itself is not an indication for termination. Teng et al. [21] reported good results after termination of pregnancy with no response to corticosteroids. The patient in the study by Shukla et al. [46] showed improvement on the second day after spontaneous abortion. However, there are many cases of successful treatment with conservative treatment and without termination of pregnancy. A significant improvement in symptoms was observed after standard treatment with HLH-1994/2004 [47], and in some cases, pregnant HLH patients were successfully treated with high-dose steroid drugs alone [8, 34, 48, 49]. A multicentre retrospective study of pregnancy-related HLH suggested that etoposide should be administered to patients who failed to respond to corticosteroids and IVIG [40]. Although there is insufficient evidence to prove that termination of pregnancy is beneficial to the remission of HLH, it should be considered if there is no response to HLH medication during pregnancy.

The decision to terminate pregnancy requires a comprehensive consideration of factors, including the maternal condition, gestational age, disease factors, and life-threatening symptoms [36]. The severity of maternal disease plays a key role. Termination of pregnancy in a highly inflammatory state may increase the burden on the organs of pregnant women. In addition, the delivery operation itself may increase the risk to the pregnant woman; for example, delivery induced DIC may lead to postpartum haemorrhage that requires rescue. From the foetal point of view, the risk of continuing pregnancy is high, and the safety of the foetus needs to be closely monitored. Gestational age should also be considered. If foetal lungs are immature, promoting foetal lung maturation treatment is appropriate because steroids are also beneficial in the treatment of HLH. Therefore, the termination of pregnancy depends on a comprehensive consideration to ensure maternal and foetal safety.

**Fig. 3:**
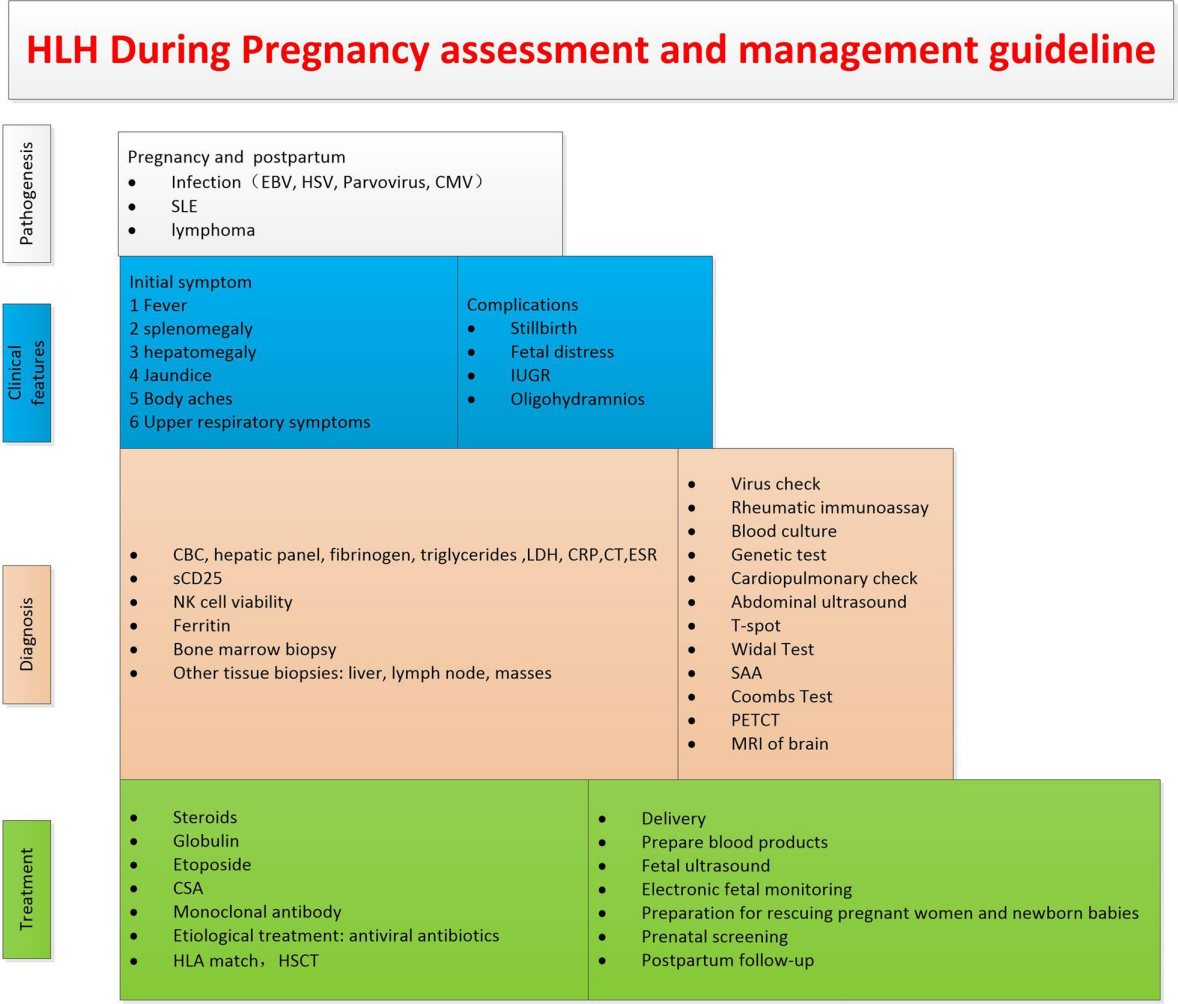
Draw a flow chart of HLH during pregnancy

In addition, in terms of the choice of the delivery mode, 23 of 55 women who terminated their pregnancies delivered by caesarean section, accounting for nearly half, as shown in Fig. 2c. In the 22 foetal death cases counted, there were eight therapeutic abortions, five vaginal deliveries, five spontaneous abortions, and four caesarean sections. In terms of puerperium management, breastfeeding can also affect the physical status of the mother, but it needs to be determined according to the patient's specific situation due to the lack of data reported (Fig. 3). All our statistical data are summarized in Additional file 1.

## Conclusions

HLH during pregnancy is a rare, fatal, and often misdiagnosed disease with a high maternal and foetal mortality rate due to its non-specific clinical manifestations. Similar to non-pregnancy, EBV infection is still the first and most important contributing factor to HLH during pregnancy. The initial clinical symptoms of HLH during pregnancy are lack of specificity. Cases with negative bone marrow biopsy and high suspicion should be considered for twice biopsy. Early diagnosis, timely treatment and good obstetric management are the necessary conditions to ensure the safety of mothers and children. Finally, termination of pregnancy requires timely and comprehensive consideration. From the perspective of obstetrics, this study enriches the comprehensive understanding of HLH diagnosis and treatment. In the future, we propose to establish a global alliance for HLH during pregnancy to standardize the collection of relevant data and form a consensus guide to optimize the diagnosis and treatment of patients.

## Supplementary Information

**Additional file 1.** Data extracted from literatures.

## Data Availability

The data used are available from the first and corresponding authors on reasonable request.

## Abbreviations

allo-HSCT: Allogeneic hematopoietic stem cell transplantation
AIHA: Autoimmune haemolytic anaemia
ALP: Alkaline phosphatase
ALT: Alanine transaminase
AST: Aspartate aminotransferase
CMV: Cytomegalovirus
CS: Caesarean section
EBV: Epstein–Barr virus
Fib: Fibrinogen
Hb: Haemoglobin
HIV: Human immunodeficiency virus
HLH: Hemophagocytic lymphohistiocytosis
HSV: Herpes simplex virus
IUGR: Intrauterine growth retardation
LDH: Lactate dehydrogenase
M-HLH: Malignancy-associated HLH
McAb: Monoclonal antibody
NK: Natural killer
PLT: Platelets
SLE: Systemic lupus erythematosus
Soluble CD25: Soluble interleukin-2 receptor
UA: Umbilical artery
VD: Vaginal delivery
VZV: Varicella zoster virus
